# Impact of Heterogeneous DNA Methylation on the Accuracy of Quantitative Methylation-Specific PCR for Detecting DNA Hypermethylation in Prostate Cancer

**DOI:** 10.3390/ijms27052249

**Published:** 2026-02-27

**Authors:** Wieke C. H. Visser, Hans de Jong, Laureen B. Janssen, Jolly Shrivastava, Peter F. A. Mulders, Jack A. Schalken, Willem J. G. Melchers

**Affiliations:** 1 Department of Product Development, Mdxhealth BV, 6534 AT Nijmegen, The Netherlands; 2 Department of Product Development, Mdxhealth, Irvine, CA 92618, USA; 3 Department of Medical Microbiology, Radboud University Medical Center, 6525 GA Nijmegen, The Netherlands; 4 Department of Urology, Radboud University Medical Center, 6525 GA Nijmegen, The Netherlands

**Keywords:** prostate cancer, DNA methylation, qMSP, liquid biopsy

## Abstract

DNA methylation is an epigenetic modification of the genome, with methylation-based biomarkers showing promise for cancer detection. Quantitative Methylation-Specific PCR (qMSP) is widely used to assess DNA methylation; however, its application to liquid biopsy samples presents technical challenges. Heterogeneity in methylation patterns may impact qMSP accuracy by reducing reliability. This study evaluated the impact of methylation heterogeneity on qMSP performance using synthetic DNA fragments mimicking methylation variation. Additionally, targeted methylation sequencing was performed on prostate cancer tissue and urine samples to examine methylation heterogeneity. The presence of unmethylated CpG sites within the primer and probe regions reduced fluorescence levels and increased Cp values, especially at the 3′-end of primers. The methylation sequencing of genomic DNA from prostate cancer tissue and urine samples revealed the presence of varying methylation patterns, correlating with qMSP outcomes. Tissue samples mainly exhibited fully methylated and fully unmethylated fragments, while urine samples consisted of a higher proportion of partly methylated fragments. The findings suggest that qMSP may not be a reliable method for methylation detection in samples with proportionally high levels of heterogeneous methylated fragments in contrast to fully methylated fragments. This highlights the need for improved DNA methylation analysis in liquid biopsy samples for clinical applications in cancer detection.

## 1. Introduction

DNA methylation is a key epigenetic modification involving the addition of a methyl group to cytosines in CpG dinucleotides. Approximately 70–80% of CpG sites in the human genome are methylated, with unmethylated CpG sites mostly clustered in CpG islands often located in gene promoter regions [1,2]. These unmethylated CpG islands regulate gene expression, and changes in DNA methylation patterns are linked to diseases, including cancer [3], making DNA hypermethylation a promising biomarker for cancer detection and prognosis [4].

Quantitative Methylation-Specific Polymerase Chain Reaction (qMSP) is a widely utilized technique for assessing the DNA methylation status of target genes [5]. qMSP utilizes two primers and a hydrolysis probe to amplify methylated regions of the targeted gene. Prior to amplification, bisulfite treatment converts unmethylated cytosines to uracils while leaving methylated cytosines intact, which enables the discrimination of methylated from unmethylated DNA.

A study by Massen et al. (2022) assessed the technical challenges inherent to qMSP-based assays and provided critical recommendations for the optimal design of methylation-specific assays [6]. Building on these insights, we previously developed several qMSP assays, including one targeting the glutathione S-transferase gene (GSTP1). GSTP1 encodes an enzyme in the glutathione S-transferase family, involved in cellular signaling pathways regulating proliferation, differentiation, and apoptosis [7]. Hypermethylation of the GSTP1 promoter region is a well-established marker of cancer, particularly in prostate cancer (PCa), where it is associated with tumorigenesis [8]. While our GSTP1 qMSP assay performed well on PCa-tissue-derived genomic DNA (gDNA), urine-derived gDNA from PCa patients showed poorly-shaped qMSP amplification curves with low fluorescence levels observed in the plateau phase of amplification. This aberrant result raised concerns about the accuracy of methylation quantification and the reliability of the assay in liquid biopsy samples.

Challenges in applying qMSP to gDNA derived from liquid biopsy materials are described earlier. DNA extracted from such samples is often degraded and present at low concentrations, significantly impacting the sensitivity and precision of the assay [9,10]. Additionally, tumor-specific epigenetic markers in liquid biopsy samples are typically present at very low allelic frequencies (<0.1%), further complicating methylation detection [11]. Furthermore, Grenaker Alnaes et al. (2015) reported that the presence of heterogeneous DNA methylation patterns in tumor tissues can lead to discrepancies between methylation quantification techniques [12]. Specifically, DNA methylation in a given region can be heterogeneous, with some CpG sites methylated and others unmethylated. This heterogeneity complicates the interpretation of qMSP results, because in qMSP, primers and the probe are specifically designed to bind fully methylated, bisulfite-converted fragments of genomic regions of interest. To achieve efficient amplification, all CpG sites covered by the primers and probe-binding sites must be methylated. As such, heterogeneous methylation increases the risk of false-negative results, as observed in breast cancer tissue, where samples that appeared unmethylated by qMSP were found to exhibit partial methylation when analyzed via pyrosequencing [12].

Despite previous studies describing the challenges associated with heterogeneous DNA methylation in the context of qMSP, its direct effect on qMSP performance, especially in liquid biopsy samples, has not been investigated. In more detail, the impact of heterogeneous DNA methylation on the qMSP amplification curve shape, including the amplification efficiency and fluorescence intensity, has not been previously reported.

In this proof-of-principle study, we aim to explore the underlying causes of the poor GSTP1 qMSP results observed with urine-derived DNA from PCa patients, defined by low fluorescence levels during amplification. First, the impact of methylation heterogeneity on the performance of GSTP1 qMSP will be evaluated by assessing how such variations influence the accuracy and reliability of methylation quantification. Secondly, the presence of heterogeneous GSTP1 methylation patterns in two small cohorts of both tissue and urine samples from PCa and benign prostate hyperplasia (BPH) patients will be examined.

## 2. Results

### 2.1. qMSP Assay

**Figure 1:**
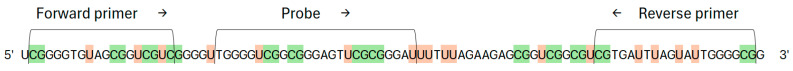
Schematic overview of the location of the qMSP assay for the GSTP1 gene. The target sequence is a methylated, bisulfite-converted sense-strand of the GSTP1 gene, located in the promoter region. Unmethylated cytosines will be converted to uracil during bisulfite treatment. Green: CpG sites, orange: unmethylated cytosines (non CpG sites) converted to uracil, sequence between brackets: primer or probe target region. G = guanine, C = cytosine, A = adenine, T = thymine, U = uracil.

The qMSP assay used in this study is designed to amplify the methylated, bisulfite-converted sense-strand GSTP1 gene. The target region is located in the gene promoter region (chromosome 11: 67.583.674–67.583.764 (hg38)). The target region covers 13 CpGs —of which 10 are covered by the primers or probe. Moreover, the target region includes 17 non-CpG-cytosines of which 10 are covered with the primers and probe (Figure 1).

### 2.2. Impact of Heterogenous Methylation on qMSP Results

To examine to what extent qMSP results are affected by the presence of heterogenous methylation patterns, synthetic double-stranded DNA fragments simulating bisulfite-converted DNA with varying methylation patterns were designed for the genomic region of GSTP1. qMSP was performed on these DNA fragments.

**Figure 2:**
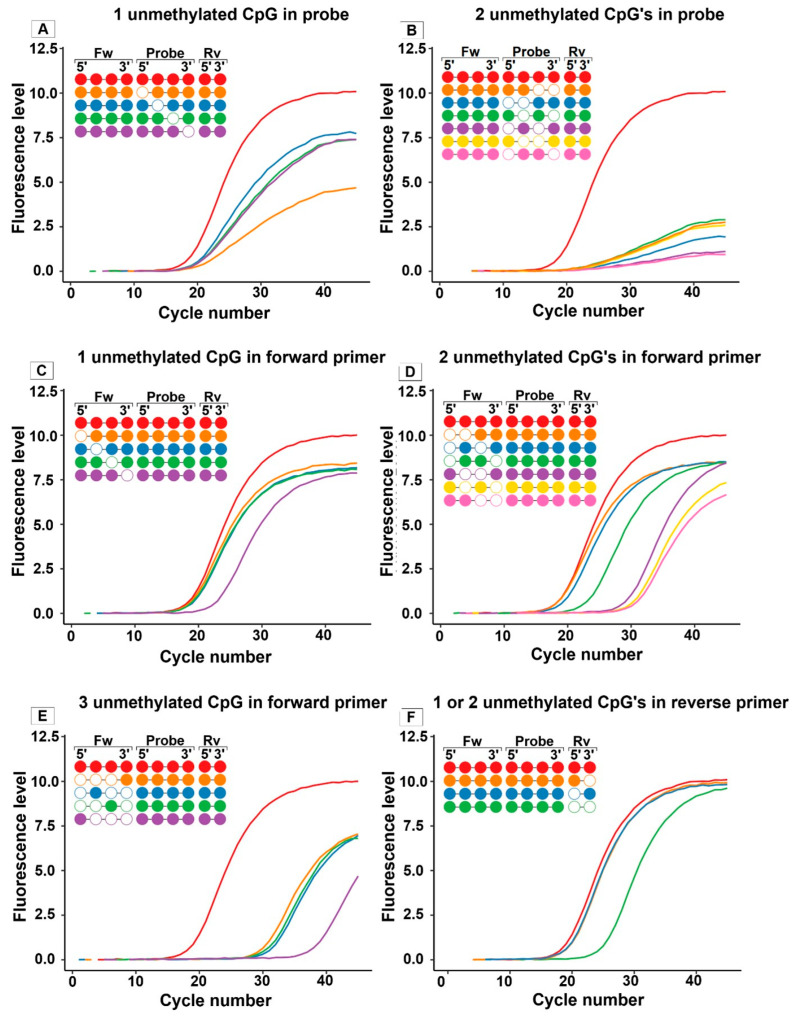
Impact of heterogeneous DNA methylation on qMSP results. qMSP was performed on designed synthetic, double-stranded DNA fragments, which are simulating bisulfite-converted DNA with varying methylation patterns for the genomic region of GSTP1. Colors of qMSP curves correspond to the methylation pattern simulated in the synthetic DNA strands. Open rounds: unmethylated state, closed rounds: methylated state. (**A**) One CpG site unmethylated in the probe region decreases fluorescence, especially when the unmethylated site is at the 5′-end of the primer, with a slight Cp increase. (**B**) Two CpG sites unmethylated in the probe region further reduce fluorescence and increase the Cp. (**C**) One CpG site unmethylated in the forward primer target region at the 3′-end increases the Cp, while an unmethylated site away from the 3′-end has less impact. (**D**) Two unmethylated CpG sites in the forward primer region have a greater impact when located close to the 3′-end, with the least effect at the 5′-end. (**E**) Three unmethylated CpG sites in the forward primer region further increase the Cp. The signal is lost when all sites are unmethylated (not shown). (**F**) For the reverse primer, unmethylation of both CpG sites increases the Cp, while one unmethylated CpG site has no effect.

The probe target region covers four CpG sites; two are located more towards the 5′-end, and the other two are located more towards the 3′-end (Figure 1). If a single CpG site in the probe’s target region was mimicking an unmethylated state (while all other CpG sites were mimicking a methylated state), a clear decrease in the endpoint fluorescence level was observed compared to fragments mimicking full methylation. The decrease in the fluorescence level is largest when this CpG site was located at most 5′-ends of the probe (Figure 2A). The shape of the qMSP curve gets worse when fluorescence levels decrease, as described before. Moreover, the Cp value increased (+1 to 1.2 Cp) as compared to fragments mimicking full methylation (Figure 2A and Table 1). When two CpG sites in the probe’s target region were designed to mimic unmethylation, fluorescence levels further decreased (Figure 2B), leading to very-poor-shaped curves, and Cp values increased (Table 1). When three or four CpG site were designed as being unmethylated, no signal was observed.

In contrast to the presence of CpG sites designed to simulate no methylation in the target region of the probe, which are mainly affecting the end-point fluorescence levels, the presence of unmethylated CpG sites in the forward primer or the reverse primer’s target regions result in a shift in the Cp value. The forward primer’s target region contains four CpG sites of which one is located at the 5′-end and the other three in the 3′-end half of the primer’s target region. When only one CpG site was simulated as unmethylated (which is not located at the 3′-end position), no effect on the qMSP result was observed. However, if this unmethylated CpG site is located at the 3′-end position, a big effect on the Cp value was observed (3.8 higher Cp value compared to fragments simulating full methylation (Figure 2C and Table 1)). Two CpG sites designed to mimic unmethylation in the forward primer’s target region further illustrate the importance of methylation of the CpG site at the 3′-end position: the closer the sites are positioned to the 3′-end, the larger the negative impact (higher Cp) on the qMSP result (Figure 2D). If one of the unmethylated CpG sites is at the most 5′-end-positioned CpG site, the impact on the Cp is less.

This reduced impact can be attributed to the fact that, in this case, the most 5′-end-positioned CpG site is followed by multiple guanines, which still allow for strong binding despite the mismatch at this 5′-end CpG site. In contrast, the other three CpG sites of the forward primer are all located at the 3′-end region, where mismatches have a more negative effect on the qMSP results (Figure 1 and Figure 2D). This is also observed when three CpG sites are designed to mimic no methylation (Figure 2E and Table 1), showing a big impact on the Cp value (>10 cycle numbers higher compared to fragments simulating full methylation) with only one CpG site designed as methylated at the 3′-end region and even 19 Cp higher with only one CpG site methylated at the most 5′-end position. All sites unmethylated in the forward primer target region resulted in almost no signal observed. 

The reverse primer’s target region covers two CpG sites, one located at the 3′-end and one located at the 5′-end. The presence of one of the two CpG sites designed as unmethylated did not significantly affect the results. However, when both CpG sites were simulated as unmethylated, a higher Cp value was observed (Figure 2F and Table 1).

Overall, the results show that the location and the number of unmethylated CpG sites affect the qMSP performance. Mismatches at the 3′-end of the forward primer region have the biggest impact, leading to a large Cp shift. For the probe, mismatches result mainly in lower fluorescence levels.

### 2.3. Presence of Heterogeneous Methylation in gDNA Derived from Both Tissue and Urine Samples from Prostate Cancer Patients

**Figure 3:**
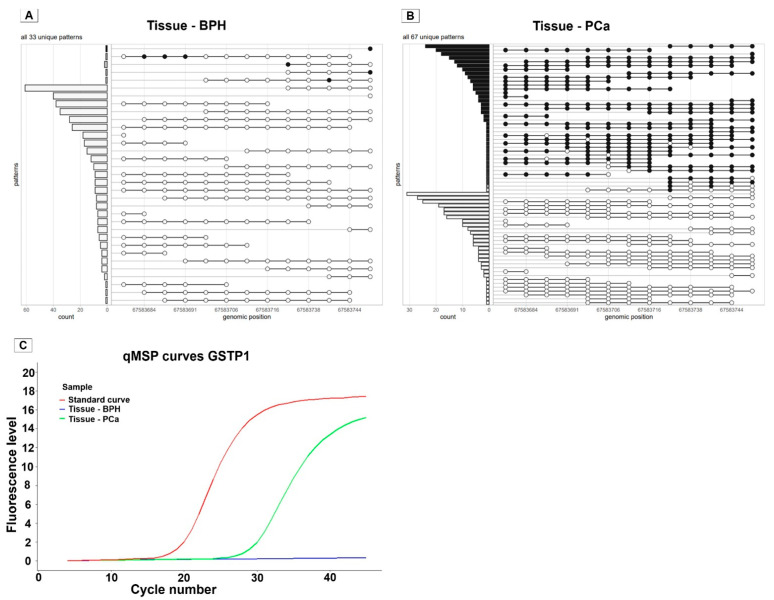
Unique GSTP1 methylation patterns detected using methylation sequencing of gDNA from BPH tissue and from PCa tissue and qMSP results. (**A**) Methylation sequencing results of gDNA from BPH tissue. Each row represents a unique methylation pattern observed for the GSTP1 target region, with filled circles showing methylated CpG sites and unfilled circles showing unmethylated CpG sites. The bar plot on the left indicates the number of reads per pattern. The BPH tissue showed a majority of unmethylated fragments. (**B**) Methylation sequencing results of gDNA from PCa tissue. In contrast to BPH, PCa tissue shows a high proportion of fully methylated GSTP1 fragments, next to partly methylated fragments. (**C**) qMSP amplification curves for GSTP1 corresponding to the BPH and PCa tissue samples shown in panels A and B, together with a standard curve for reference. The BPH sample shows no amplification detected. The PCa sample, shows a clear and well-shaped amplification curve, comparable to the standard curve. 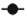: methylated CpG site, 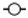: unmethylated CpG site. BPH: benign prostate hyperplasia, PCa: prostate cancer. qMSP: Quantitative Methylation-Specific qPCR.

To investigate the occurrence of heterogeneous methylation patterns and its effect on the qMSP results, gDNA derived from both tissue and urine samples from prostate cancer patients were evaluated using both methylation sequencing and qMSP. After preprocessing the sequencing data, the epialleleR package is used in Rstudio to extract methylation patterns detected in sequenced DNA fragments. The resulting graphs show all unique patterns detected within the GSTP1 amplicon region, varying in the methylation pattern and fragment length and their observed frequencies (Figure 3A,B).

In the gDNA sample derived from BPH tissue, most patterns were fully unmethylated, with variation in the fragment size and the genomic regions covered. A small number of reads did contain one or more methylated sites; however, their frequency was low (1–2 fragments) as compared to the abundance of unmethylated fragments (Figure 3A). This BPH sample was shown to be negative based on qMSP (Figure 3C). In contrast, the sample with gDNA from PCa tissue showed many fully methylated patterns. Many patterns observed in gDNA from PCa tissue were fully methylated, with variation in the fragment size and covered regions. Additionally, some fragments were partly methylated (with one CpG site unmethylated), but these patterns were less abundant than the fully methylated patterns (Figure 3B). The qMSP curve for this sample showed a clear positive signal with a well-shaped amplification curve (Figure 3C).

**Figure 4:**
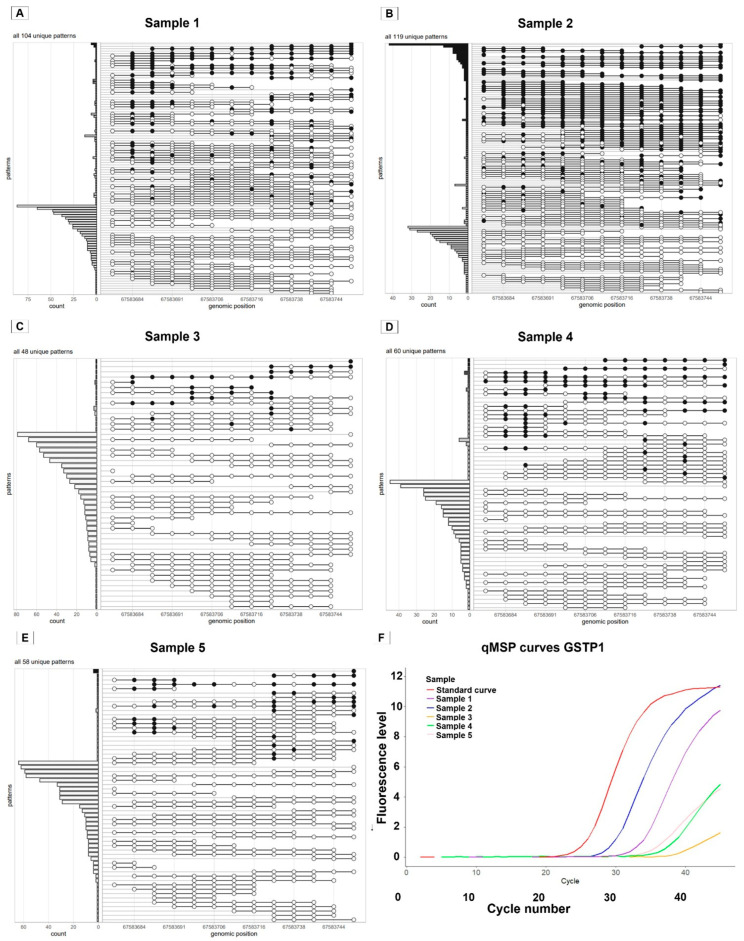
Unique GSTP1 methylation patterns detected using methylation sequencing of gDNA from PCa urine and qMSP results. (**A**–**E**) Methylation sequencing results of gDNA isolated from urine samples of five prostate cancer patients (samples 1–5). Each row shows a unique methylation pattern observed across the GSTP1 target region, with filled circles indicating methylated CpG sites and unfilled circles indicating unmethylated CpG sites. The bar plot on the left shows the number of reads per pattern. Sample 2 (panel **B**) shows the highest amount of fully methylated fragments, followed by sample 1. Both samples show, next to the presence of fully methylated fragments, also partly methylated fragments and many fully unmethylated fragments. Samples 3–5 show no or almost no fully methylated fragments, some partly methylated fragments, and many unmethylated fragments. (**F**) qMSP amplification curves for GSTP1 corresponding to samples 1–5, shown together with a standard curve for reference. Sample 1 and 2 show a clear and well-shaped amplification curve. This is consistent with the presence of fully methylated GSTP1 fragments observed via methylation sequencing (panels **A**,**B**). In contrast, samples 3–5 show poorly shaped amplification curves, corresponding to the absence of fully methylated fragments in the sequencing data (panel **C**–**E**). 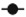: methylated CpG site, 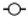: unmethylated CpG site. qMSP: Quantitative Methylation-Specific qPCR.

Following this, DNA derived from the urine of men with PCa was examined using the same tools as described above (Figure 4). A clear difference in the methylation pattern is observed comparing the patterns from urinary DNA (Figure 4A–E) to PCa tissue gDNA (Figure 3B): more unique patterns that are partly methylated are observed in urinary DNA. Urinary samples 1 and 2 (Figure 4A,B) show both the presence of fully methylated fragments, as well as partly methylated fragments. qMSP curves are well-shaped for both samples. In contrast, urinary samples 3, 4, and 5 show poor qMSP curves with low end-point fluorescence levels. Those samples show some partly methylated fragments and (almost) no fully methylated fragments for this region.

## 3. Discussion

We evaluated the effect of heterogeneous methylation on qMSP by applying this method to synthetic DNA fragments designed to mimic bisulfite-converted DNA, containing varying patterns of methylated and unmethylated CpG sites. This study focuses on a single, well-established prostate cancer methylation target, GSTP1. Our analysis revealed that the heterogeneous methylation notably alters the shape of GSTP1 qMSP curves and corresponding Cp values. Furthermore, we examined the presence of heterogeneous methylation patterns in DNA extracted from tissue and urine from a small cohort of PCa patients using methylation sequencing. Following this, qMSP targeting GSTP1 was applied to these samples to investigate the association between poor-shaped qMSP curves and the identified methylation patterns. Sequencing results confirmed the presence of heterogeneous methylation of the GSTP1 target in both tissue and urine, and moreover, gDNA samples with relatively high amounts of heterogenous methylated GSTP1 fragments present poor-shaped qMSP curves. This finding underscores a critical limitation: poorly shaped qMSP curves can compromise the accuracy of Cp value determination and subsequent methylation quantification. Overall, these results show that there is a potential limitation of qMSP for the methylation analysis of targets showing heterogeneous methylation patterns, such as GSTP1, which may affect the reliability of Cp value determination.

The GSTP1 qMSP results based on synthetic DNA fragments showed that the genomic location of one or more unmethylated CpG(s), and therefore the genomic location of the mismatch between DNA template and primer or probe, is playing a major role in the subsequent effect on the qMSP result. Mismatches in the probe target region mainly reduce fluorescence and, in some variants, also increase the Cp value, especially when unmethylated CpG sites are at the 5′-end of the target region. For the forward primer region, mismatches at the 3′-end of the primer target region have the most significant impact on the Cp, while mismatches near the 5′-end have a minimal effect. Increasing the number of mismatches in the forward primer results in a larger impact on the results, with no signal detected when all sites are unmethylated. In the reverse primer region, one unmethylated CpG has no effect, but two unmethylated CpG sites significantly increase the Cp. The difference in the effect of a mismatch in a primer or probe can be explained by their function in the qMSP reaction. A mismatch in the primer, especially at the 3′-end, decreases the amplification efficiency due to reduced polymerase activity, while a mismatch in the probe region is associated with decreased fluorescence detection rather than the amplification efficiency.

Additionally, the nature of the bases flanking the mismatch influences its impact on the GSTP1 qMSP outcome. When the mismatch is adjacent to guanine bases, the impact of the mismatch is minimal. This can be explained by the fact that the guanine bases will ensure strong template-primer (or template-probe) binding, independent of the mismatch. The importance of the genomic location of the mismatch was observed before. Other researchers recommend designing primers so that a CpG site is located at the 3′-end of the primer target regions. This will decrease the risk of the amplification of unmethylated or unconverted DNA fragments [6,13,14]. Other important technical aspects of efficient qMSP assays are the use of an optimal annealing temperature and MgCl2 concentration [15]. One limitation of this study is that each GSTP1 qMSP reaction using synthetic DNA fragments examined only a single variant. However, sequencing data from PCa urine and tissue samples show the presence of multiple GSTP1 epialleles varying in methylation levels. Future research should investigate the effect of heterogeneous methylation on qMSP by testing various mixtures of different epialleles thereby more accurately simulating the methylation diversity observed in PCa samples. Importantly, the composition and relative abundance of these epiallele mixtures should be based on methylation patterns observed in clinical samples. A limitation of this study is that technical replicates were not performed for each measurement; therefore, statistical summaries of Cp shifts and reductions in fluorescence levels could not be described. Future studies should include this in the study design. In addition, the current paper only included experiments using one target gene (GSTP1). To generalize the findings, additional target genes should be investigated, ideally covering a range of CpG densities and different CpG sequence contexts.

The comparison of sequencing results and GSTP1 qMSP data showed that DNA samples exhibiting poorly shaped qMSP curves also contained relatively high proportions of heterogeneous methylated GSTP1 DNA fragments. This finding highlights the association between the curve morphology and the presence of heterogeneous methylation and supports our hypothesis. However, it is important to note that poorly shaped curves are not a direct indicator of heterogeneous methylation, as other factors (DNA degradation, the presence of PCR inhibitors, or incomplete bisulfite conversion) can also result in aberrant MSP curve morphology.

Another interesting observation is the difference in the ratio of fully methylated to partly methylated fragments detected in tissue compared to urine. Tissue samples predominantly displayed fully methylated GSTP1 fragments, whereas some urine samples contained only partly methylated fragments, despite the expectation of fully methylated fragments in the urine. This discrepancy could be attributed to the acidic conditions under which PCa urine samples were collected, potentially causing deamination of methylated cytosines into uracil and increasing the likelihood of false-negative methylation signals [16]. Another potential explanation for the discrepancy is that the partly methylated GSTP1 fragments observed in urine may not originate from prostate (cancer) tissue. Urine DNA is a mixture of DNA fragments derived from both normal and tumor cells, and therefore, the methylation profiles observed in urine DNA may not directly reflect the methylation patterns observed in prostate tissue. These findings make it clear that a careful interpretation of qMSP data is required when analyzing methylation levels in liquid biopsy samples, especially when collected in an acid preservative. Moreover, it is unknown if the copies detected via qMSP are originating from fully- or partly methylated fragments, making interpretation difficult.

In contrast to qMSP, methylation sequencing offers a major advantage in detecting and analyzing epialleles and heterogenous methylation patterns. Aberrant methylation could be an early sign of PCa carcinogenesis, emphasizing the potential clinical value of detecting partly methylated fragments. This is also described by Curradi et al. [16]. They investigated the role of methylated CpG sites in inhibition of transcription and showed that only a few methylated CpG sites can already result in the inhibition of transcription. Only three CpG sites methylated resulted in a 45% reduction in transcription, and the reduction was larger with more than three sites methylated. They also demonstrated that the level of inhibition was also associated with the distance between the methylated CpG sites and the promoter region [16]. Next to that, Gupta et al. (2023) showed that the level of GSTP1 promoter methylation is correlated with prostate cancer progression [17]. Therefore, further investigation into the clinical relevance of specific methylation patterns is needed.

A notable disadvantage of this DNA methylation sequencing technique is that a fragmentation step is incorporated during library preparation. As a result, DNA fragments covering the full GSTP1 qMSP target region might have been present in the qMSP reaction, whereas those fragments might have been fragmented in the methylation sequencing reaction. Also, the qMSP technique is more sensitive for methylation detection as compared to the methylation sequencing tool, partly due to the limited input amount of gDNA for methylation detection (200 ng) as compared to qMSP (20 µL ranging from 20 to 800 ng). Higher sensitivity with qMSP is also observed in our results, for example sample 4 showed 41 methylated copies based on qMSP in contrast to 0–10 copies in sequencing. Next to that, another major limitation of methylation sequencing is that a very high depth (up to 100,000×) is required to detect low-abundance methylated fragments [18].

Another critical factor of methylation detection that can affect the reliability of the results is the conversion efficiency of unmethylated cytosines prior to methylation detection. Bisulfite conversion is the golden standard used for methylation detection, also used in our research prior to qMSP. Incomplete conversion can result in false-positive methylation results [19]. However, an alternative enzymatic conversion technique, as used in the sequencing experiments for this paper, which has been shown to offer higher conversion rates and DNA yields, is increasingly being used in methylation sequencing applications [20].

Since both qMSP and methylation sequencing have important limitations, and further research on methylation detection should use other tools for methylation detection in urine, for example digital droplet PCR, which incorporates a dilution and a pre-amplification step. This combination was shown to improve the sensitivity of methylation detection in a large background of unmethylated fragments [15]. Other advantages of this technique are that the reaction is less prone to PCR inhibitors and that it enables the absolute quantification of target DNA without the need for standard curves. However, this technology has limitations as well. A limited number of CpG sites can be analyzed simultaneously, as specific primers and probes need to be designed for each epiallele. Moreover, pre-amplification steps might introduce bias in your reaction, negatively impacting your results.

Overall, our findings show that qMSP may not be a reliable tool for methylation detection in liquid biopsy samples with proportionally high levels of heterogeneous methylated DNA fragments in contrast to fully methylated fragments. Importantly, these results were observed for the GSTP1 target analyzed in this study, and the extrapolation of these results to other targets should be done with caution. The generalizability is probably affected by locus-specific factors such as the sequence context, primer and probe design, and CpG density. Despite the known limitations of qMSP in the context of heterogeneous methylation, our study provides detailed insight into this limitation. We analyzed, in detail, how changes in methylation patterns and sequence mismatches at specific CpG sites within the GSTP1 target affect qMSP amplification and fluorescence signals. These findings can help other researchers in designing and optimizing primers and probes for qMSP assays. As this is a proof-of-principle study, larger validation of this concept is required.

## 4. Materials and Methods

### 4.1. Design of eBLOCK with Known Variation in Methylation

Synthetic double-stranded DNA fragments simulating varying DNA methylation patterns were designed and ordered (eBlocks, Integrated DNA Technologies, Leuven, Belgium); see Supplementary Table S1. eBlocks are short, double-stranded DNA fragments ranging from 300 to 1500 base pairs, in which individual bases can be modified. eBlocks were designed to simulate the sequence of bisulfite-converted DNA of a region of interest with varying methylation patterns. Variations in DNA methylation patterns were introduced at genomic positions targeted by qMSP primers and probes for the gene GSTP1. The eBlocks were designed as a simplified experimental approach for the purpose of this study and do not cover all methylation heterogeneity observed in clinical samples.

### 4.2. qMSP on eBLOCKs

qMSP was performed on eBLOCKs using a duplex qMSP assay targeting the genes GSTP1 and Beta-actin (ACTB) on a LightCycler 480 Instrument (Roche Life Sciences, Indianapolis, IN, USA). The reaction mixture consisted of 6 µL eBLOCK (ranging from 80–800,000 copies of target fragment), 0.4 µM of each primer (Integrated DNA Technologies, Leuven, Belgium), 0.1 µM of each probe (Integrated DNA Technologies, Leuven, Belgium), 4 µL 5× concentrated PerfeCTa MultiPlex qPCR Toughmix (cat. #95147, Quantabio, Beverly, MA, USA), and nuclease-free water. The qPCR program consisted of an initial denaturation step at 95 °C for 3 min, followed by an amplification step of 15 s at 94 °C, annealing/elongation at 64 °C for 60 s, and subsequently a cooling step at 37 °C for 2 min. Cp values were converted to copy numbers using a reference curve. Following this, copy numbers were normalized for input by dividing the copy numbers of the genes of interest over ACTB copy numbers. The % GSTP1 ratio compared to fully methylated was calculated as the GSTP1 ratio detected in the specific methylation pattern/GSTP1 ratio detected in the fully methylated sample × 100%.

### 4.3. Sample Collection

To explore the presence of heterogeneous methylation patterns and their impact on the qMSP curve, genomic DNA obtained from tissue and urine samples of prostate cancer patients was analyzed using both methylation sequencing and qMSP. Urine samples were initially collected from men with low-grade prostate cancer who participated in a prospective, multi-center study in the USA. For the present study, only residual urine samples and corresponding anonymized clinical data were used, in accordance with participants’ prior consent for future research use. Following a digital rectal exam (DRE) under routine practice by urologists, 10–20 mL of first-void urine was collected using a urine collection device (Novosanis, Wijnegem, Belgium). The urine collection tube contained a preservative to ensure stability of the nucleic acids. Subsequently, samples were shipped to Mdxhealth on room temperature and stored at −80 °C until use.

Fresh-frozen tissue from one patient with benign prostatic hyperplasia (BPH) and one patient diagnosed with prostate cancer (ISUP grade 3) was used to isolate genomic DNA (gDNA). The fresh-frozen tissue samples were obtained from the biobank of the Department of Urology, Radboudumc, Nijmegen, The Netherlands. These archived samples were collected between the 1980s and 2000s under an opt-out regulation and were used in accordance with institutional guidelines for secondary use of anonymized biobank material. Data from the biobank and urine sample study were accessed for research purposes between 1 December 2024 and 11 October 2025. All data used in this study were fully anonymized prior to analysis, and none of the authors had access to information that could identify individual participants during or after data collection.

### 4.4. DNA Extraction and Quantification

DNA was extracted from 10 mL of urine using the Heater Shaker Magnet 2.0 Instrument (HSM, Promega Corporation, Madison, WI, USA) to concentrate large-volume urine samples prior to extraction, followed by extraction using the Maxwell RSC Instrument (Promega Corporation, Madison, WI, USA), with the Maxwell RSC miRNA Plasma and Serum Kit (cat. no. AS1460, Promega Corporation, Madison, WI, USA) with a protocol optimized for urine. First, 10 mL of urine was transferred to a 50 mL tube, mixed with 3.7 mL Cell Lysis Buffer (CLB, cat. no. A1731, Promega Corporation, Madison, WI, USA) and 250 µL Proteinase K (20 mg/mL, cat. no. A5051, Promega Corporation, Madison, WI, USA), and incubated at 37 °C for 38 min with shaking at 500 rpm. Then, 8 mL of isopropanol was added. All resin (containing magnetic beads) from position 2 of the kit cartridge was resuspended and added to the mixture, followed by incubation at 25 °C for 60 min with shaking at 500 rpm. Next, the magnetic beads with bound DNA were collected at 25 °C for 10 min using the magnet in the instrument. The supernatant was then removed, while saving 500 µL per sample. The samples were removed from the instrument, and the resin was resuspended in the saved 500 µL of supernatant. Next, 500 µL of isopropanol (from position 1 of the kit cartridge) and 230 µL CLB were added. After mixing, the mixture was transferred to position 1 of the kit cartridge. DNA extraction continued using the Maxwell RSC Instrument following the kit protocol. A volume of 65 µL elution buffer (nuclease-free water) was added to the elution tubes. After completion of the protocol, sample volumes were ~45 µL.

After DNA extraction, DNA quantification was performed using the Qubit fluorometer (Thermo Fisher Scientific, Waltham, MA, USA) with the Qubit dsDNA High Sensitivity (HS) Assay Kit for urine samples and the Qubit dsDNA Broad Range (BR) Assay Kit for tissue samples following the manufacturer’s protocol.

### 4.5. Library Preparation

Following this, 50 ng to 200 ng from each gDNA sample was fragmented into approximately 240–290 base pair fragments using the Covaris S2 Focused Ultrasonicator (Covaris Inc., Woburn, MA, USA). Methylation sequencing libraries were prepared using the NEBNext Enzymatic Methyl-seq Kit (Cat. no. 101976, New England Biolabs, Ipswich, MA, USA), the Twist Fast Hybridization and Wash Kit with amp mix (Cat. no. 104180, Twist Bioscience, South San Francisco, CA, USA), Twist Universal Blockers (Cat. no. 100578, Twist Bioscience, South San Francisco, CA, USA), Twist Binding and Purification Beads (Cat. no. 100983, Twist Bioscience, South San Francisco, CA, USA), and Twist Methylation Enhancer (Cat. no. 103557, Twist Bioscience, South San Francisco, CA, USA). Library preparation was performed according to the manufacturer’s protocols. The targeted panel (625 Kbp) consisted of 5402 probes designed to capture methylated and unmethylated DNA from 366 regions of interest. DNA regions of interest were selected from the literature (associated with prostate cancer) or derived from internal, unpublished findings. Pooled libraries (200–560 ng) were sequenced on a NovaSeq6000 instrument (Illumina, Inc., San Diego, CA, USA) by Genomescan (Leiden, The Netherlands) following standard sequencing protocols.

### 4.6. Methylation Sequencing Data Preprocessing and Analysis

Methylation sequencing raw reads were pre-processed, starting with using the FastQC package (v0.12.1) to identify the read quality, adapter content, and sequence lengths. Cutadapt (v3.4) was used to trim adapter sequences and bad quality bases. Filtered reads were aligned to the reference genome (hg38) using Bismark (v0.22.3). The generated bam files were used as input for methylation calling using the EpialleleR package (v1.14.0) in RStudio. Alignments were sorted based on read names instead of coordinates using Samtools (v1.19). Name-sorted alignments were provided to the EpialleleR package, in which the extractPatterns function (with settings clip.patterns = TRUE and the GSTP1 assay region specified as input for the BED file) was used to extract DNA methylation patterns. Following this, the plotPatterns function was used to visualize the methylation patterns (settings npatterns.per.bin = Inf, marginal = “count”, genomic.scale = “discrete”, tag = NULL, plot = TRUE).

### 4.7. Bisulfite-Treatment and qMSP on Urine and Tissue Samples

For each tissue and urine sample, 2 µL (gDNA from tissue) or 20 µL (gDNA from urine) of extracted DNA was inserted into a bisulfite conversion reaction using the EZ DNA Methylation-Lightning Kit (Zymo Research, Irvine, CA, USA) with elution in 10.5 µL (gDNA from tissue) or 12 µL (gDNA from urine) of elution buffer. Next, methylation levels of the target genes GSTP1 and ACTB (reference gene) in the urinary- and tissue-derived bisulfite-converted DNA (BT-DNA) samples were quantified using a duplex qMSP assay on a LightCycler 480 Instrument (Roche Life Sciences, Indianapolis, IN, USA). The reaction mixture consisted of 2 µL (BT-DNA from tissue) or 10 µL (BT-DNA from urine) of bisulfite-treated gDNA, 0.4 µM of each primer, 0.1 µM of each probe, 4 µL of 5× concentrated PerfeCTa MultiPlex qPCR Toughmix (cat. no. 95147, Quantabio, Beverly, MA, USA), and nuclease-free water. The qPCR program consisted of an initial denaturation step at 95 °C for 3 min, followed by an amplification step of 15 s at 94 °C, annealing/elongation at 64 °C for 60 s, and subsequently a cooling step at 37 °C for 2 min. Cp values were converted to copy numbers using a reference curve. Following this, copy numbers were normalized for input by dividing the copy numbers of the genes of interest over ACTB copy numbers.

## Figures and Tables

**Table 1:** Impact of unmethylated CpG sites in target region on the fluorescence levels, Cp value, and GSTP1 ratio.

Methylation Pattern Description	Methylation Pattern	Change in End-Point Fluorescence Levels (%)	Average Shift in Cp	% GSTP1 Ratio
Fully methylated	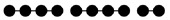	0%	0	100%
Fully methylated	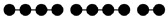	0%	0	100%
1 unmethylated CpG site in probe	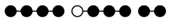	−53.59%	−1.23	38%
1 unmethylated CpG site in probe	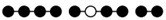	−23.37%	−1.02	58%
1 unmethylated CpG site in probe	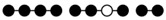	−26.85%	−1.17	43%
1 unmethylated CpG site in probe	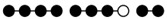	−26.71%	−1.26	40%
2 unmethylated CpG sites in probe	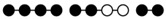	−72.72%	−3.38	11%
2 unmethylated CpG sites in probe	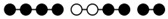	−80.87%	−3.45	10%
2 unmethylated CpG sites in probe	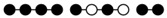	−71.31%	−2.67	17%
2 unmethylated CpG sites in probe	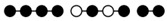	−88.98%	−5.14	2%
2 unmethylated CpG sites in probe	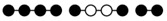	−74.35%	−3.81	9%
2 unmethylated CpG sites in probe	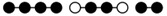	−90.66%	No Cp	0%
3 unmethylated CpG sites in probe	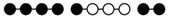	No signal	No signal	0%
3 unmethylated CpG sites in probe	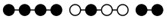	No signal	No signal	0%
3 unmethylated CpG sites in probe	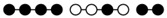	No signal	No signal	0%
3 unmethylated CpG sites in probe	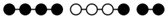	No signal	No signal	0%
4 unmethylated CpG sites in probe	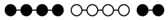	No signal	No signal	0%
1 unmethylated CpG site in fw primer	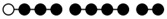	−15.78%	0.20	116%
1 unmethylated CpG site in fw primer	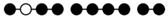	−18.29%	−0.25	101%
1 unmethylated CpG site in fw primer	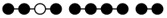	−19.27%	−0.04	77%
1 unmethylated CpG site in fw primer	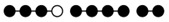	−21.31%	−3.83	7%
2 unmethylated CpG sites in fw primer	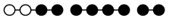	−15.34%	0.41	85%
2 unmethylated CpG sites in fw primer	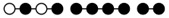	−15.16%	−0.50	43%
2 unmethylated CpG sites in fw primer	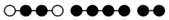	−15.67%	−4.24	3%
2 unmethylated CpG sites in fw primer	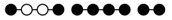	−15.66%	−10.50	0%
2 unmethylated CpG sites in fw primer	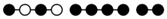	−26.69%	−11.44	0%
2 unmethylated CpG sites in fw primer	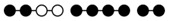	−33.42%	−11.74	0%
3 unmethylated CpG sites in fw primer	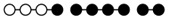	−53.14%	−10.97	0%
3 unmethylated CpG sites in fw primer	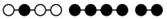	−29.54%	−12.30	0%
3 unmethylated CpG sites in fw primer	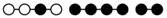	−30.38%	−11.94	0%
3 unmethylated CpG sites in fw primer	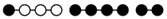	−32.08%	−19.14	0%
4 unmethylated CpG sites in fw primer	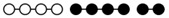	−61.02%	−19.50	0%
1 unmethylated CpG site in rv primer	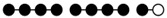	−1.67%	−0.71	86%
1 unmethylated CpG site in rv primer	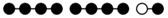	−2.67%	−0.65	68%
2 unmethylated CpG sites in rv primer	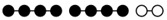	−4.71%	−6.08	2%

## Data Availability

Data cannot be shared publicly because they contain information derived from human samples obtained from institutional biobanks and clinical studies subject to privacy and data protection regulations.

## Supplementary Materials

The following supporting information can be downloaded at: https://www.mdpi.com/article/10.3390/ijms27052249/s1.

## Abbreviations

The following abbreviations are used in this manuscript:

ACTB	Beta-actin (reference gene)
BAM	Binary Alignment Map
BPH	Benign Prostate Hyperplasia
BT-DNA	Bisulfite-treated DNA
CpG	Cytosine-phosphate-Guanine dinucleotide
Cp	Crossing point
DRE	Digital Rectal Exam
eBlock	Synthetic double-stranded DNA fragment (Integrated DNA Technologies)
FastQ	Text-based format for storing biological sequence data with quality scores
gDNA	Genomic DNA
GSTP1	Glutathione S-transferase Pi 1
HSM	Heater Shaker Magnet 2.0 Instrument
ISUP	International Society of Urological Pathology (grading system)
PCa	Prostate Cancer
PCU	Prostate Cancer Urine study
qMSP	Quantitative Methylation-Specific Polymerase Chain Reaction

## References

[1] Ndlovu M.N., Denis H., Fuks F. (2011). Exposing the DNA methylome iceberg. Trends Biochem. Sci..

[2] Jang H.S., Shin W.J., Lee J.E., Do J.T. (2017). CpG and non-CpG methylation in epigenetic gene regulation and brain function. Genes.

[3] Suzuki M.M., Bird A. (2008). DNA methylation landscapes: Provocative insights from epigenomics. Nat. Rev. Genet..

[4] Mikeska T., Craig J.M. (2014). DNA methylation biomarkers: Cancer and beyond. Genes.

[5] Ramalho-Carvalho J., Henrique R., Jerónimo C. (2018). Methylation-specific PCR. DNA Methylation Protocols.

[6] Massen M., Lommen K., Wouters K.A.D., Vandersmissen J., Van Criekinge W., Herman J.G., Melotte V., Schouten L.J., van Engeland M., Smits K.M. (2022). Technical considerations in PCR-based assay design for diagnostic DNA methylation cancer biomarkers. Clin. Epigenet..

[7] Martignano F., Gurioli G., Salvi S., Calistri D., Costantini M., Gunelli R., De Giorgi U., Foca F., Casadio V. (2016). GSTP1 methylation and protein expression in prostate cancer: Diagnostic implications. Dis. Markers.

[8] Costa V.L., Henrique R., Jerónimo C. (2007). Epigenetic markers for molecular detection of prostate cancer. Dis. Markers.

[9] Hulbert A., Jusue-Torres I., Stark A., Chen C., Rodgers K., Lee B., Griffin C., Yang A., Huang P., Wrangle J. (2017). Early detection of lung cancer using DNA promoter hypermethylation in plasma and sputum. Clin. Cancer Res..

[10] Diaz L.A., Bardelli A. (2014). Liquid biopsies: Genotyping circulating tumor DNA. J. Clin. Oncol..

[11] Ilie M., Hofman V., Long E., Bordone O., Selva E., Washetine K., Marquette C.H., Hofman P. (2014). Current challenges for detection of circulating tumor cells and cell-free circulating nucleic acids, and their characterization in non-small cell lung carcinoma patients. What is the best blood substrate for personalized medicine?. Ann. Transl. Med..

[12] Grenaker Alnaes G.I., Ronneberg J.A., Kristensen V.N., Tost J. (2015). Heterogeneous DNA methylation patterns in the GSTP1 promoter lead to discordant results between assay technologies and impede its implementation as epigenetic biomarkers in breast cancer. Genes.

[13] Herman J.G., Graff J.R., Myöhänen S., Nelkin B.D., Baylin S.B. (1996). Methylation-specific PCR: A novel PCR assay for methylation status of CpG islands. Proc. Natl. Acad. Sci. USA.

[14] Wojdacz T.K., Hansen L.L., Dobrovic A. (2008). A new approach to primer design for the control of PCR bias in methylation studies. BMC Res. Notes.

[15] Menschikowski M., Jandeck C., Friedemann M., Nacke B., Hantsche S., Tiebel O., Sukocheva O., Hagelgans A. (2018). Identification of rare levels of methylated tumor DNA fragments using an optimized bias based pre-amplification-digital droplet PCR (OBBPA-ddPCR). Oncotarget.

[16] Curradi M., Izzo A., Badaracco G., Landsberger N. (2002). Molecular mechanisms of gene silencing mediated by DNA methylation. Mol. Cell. Biol..

[17] Gupta H., Inoue H., Nakai Y., Nakayama M., Jones T., Hicks J.L., Kumar B., Gurel M., Nelson W.G., De Marzo A.M. (2023). Progressive spreading of DNA methylation in the GSTP1 promoter CpG island across transitions from precursors to invasive prostate cancer. Cancer Prev. Res..

[18] Liu B., Ricarte Filho J., Mallisetty A., Villani C., Kottorou A., Rodgers K., Chen C., Ito T., Holmes K., Gastala N. (2020). Detection of promoter DNA methylation in urine and plasma aids the detection of non–small cell lung cancer. Clin. Cancer Res..

[19] Kristensen L.S., Raynor M., Candiloro I., Dobrovic A. (2012). Methylation profiling of normal individuals reveals mosaic promoter methylation of cancer-associated genes. Oncotarget.

[20] Vaisvila R., Ponnaluri V.K.C., Sun Z., Langhorst B.W., Saleh L., Guan S., Dai N., Campbell M.A., Sexton B.S., Marks K. (2021). Enzymatic methyl sequencing detects DNA methylation at single-base resolution from picograms of DNA. Genome Res..

