# Supplementary material for: Impact of Heterogeneous DNA Methylation on the Accuracy of Quantitative Methylation-Specific PCR for Detecting DNA Hypermethylation in Prostate Cancer
Source: Int J Mol Sci. 2026 Feb 27;27(5):2249. doi: 10.3390/ijms27052249 (PMC12984612; doi:10.3390/ijms27052249)
Supplement: Supplementary file 1 [file ijms-27-02249-s001.zip › ijms-4098168-supplementary.pdf]

Table S1– Design of eBLOCK

**Variant name**	**Variant methylation pattern**<br>→ Forward (5' 3') → Probe (5' 3') ← Reverse (5' 3')	**Sequence (5'- end to 3'-end)**
Fully methylated	●●●● ●●●● ●●	acgttcgggtgtagcggtcgtcgggttggggtcggcgggagttcgcgggattttttagaagagcggtcggcgtcgtgatttagtattggggcggagcgg
1 unmethylated CpG in probe	●●●● ○●●● ●●	acgttcgggtgtagcggtcgtcgggttggggttggcgggagttcgcgggattttttagaagagcggtcggcgtcgtgatttagtattggggcggagcgg
1 unmethylated CpG in probe	●●●● ●○●● ●●	acgttcgggtgtagcggtcgtcgggttggggtcggtgggagttcgcgggattttttagaagagcggtcggcgtcgtgatttagtattggggcggagcgg
1 unmethylated CpG in probe	●●●● ●●○● ●●	acgttcgggtgtagcggtcgtcgggttggggtcggcgggagtttgcgggattttttagaagagcggtcggcgtcgtgatttagtattggggcggagcgg
1 unmethylated CpG in probe	●●●● ●●●○ ●●	acgttcgggtgtagcggtcgtcgggttggggtcggcgggagttcgtgggattttttagaagagcggtcggcgtcgtgatttagtattggggcggagcgg
2 unmethylated CpG's in probe	●●●● ●●○○ ●●	acgttcgggtgtagcggtcgtcgggttggggtcggcgggagtttgtgggattttttagaagagcggtcggcgtcgtgatttagtattggggcggagcgg
2 unmethylated CpG's in probe	●●●● ○○●● ●●	acgttcgggtgtagcggtcgtcgggttggggttggtgggagttcgcgggattttttagaagagcggtcggcgtcgtgatttagtattggggcggagcgg
2 unmethylated CpG's in probe	●●●● ●○●○ ●●	acgttcgggtgtagcggtcgtcgggttggggtcggtgggagttcgtgggattttttagaagagcggtcggcgtcgtgatttagtattggggcggagcgg
2 unmethylated CpG's in probe	●●●● ○●○● ●●	acgttcgggtgtagcggtcgtcgggttggggttggcgggagtttgcgggattttttagaagagcggtcggcgtcgtgatttagtattggggcggagcgg
2 unmethylated CpG's in probe	●●●● ●○○● ●●	acgttcgggtgtagcggtcgtcgggttggggtcggtgggagtttgcgggattttttagaagagcggtcggcgtcgtgatttagtattggggcggagcgg
2 unmethylated CpG's in probe	●●●● ○●●○ ●●	acgttcgggtgtagcggtcgtcgggttggggttggcgggagttcgtgggattttttagaagagcggtcggcgtcgtgatttagtattggggcggagcgg
3 unmethylated CpG's in probe	●●●● ●○○○ ●●	acgttcgggtgtagcggtcgtcgggttggggtcggtgggagtttgtgggattttttagaagagcggtcggcgtcgtgatttagtattggggcggagcgg
3 unmethylated CpG's in probe	●●●● ○●○○ ●●	acgttcgggtgtagcggtcgtcgggttggggttggcgggagtttgtgggattttttagaagagcggtcggcgtcgtgatttagtattggggcggagcgg
3 unmethylated CpG's in probe	●●●● ○○●○ ●●	acgttcgggtgtagcggtcgtcgggttggggttggtgggagttcgtgggattttttagaagagcggtcggcgtcgtgatttagtattggggcggagcgg
3 unmethylated CpG's in probe	●●●● ○○○● ●●	acgttcgggtgtagcggtcgtcgggttggggttggtgggagtttgcgggattttttagaagagcggtcggcgtcgtgatttagtattggggcggagcgg
4 unmethylated CpG's in probe	●●●● ○○○○ ●●	acgttcgggtgtagcggtcgtcgggttggggttggtgggagtttgtgggattttttagaagagcggtcggcgtcgtgatttagtattggggcggagcgg
1 unmethylated CpG in forward primer	○●●● ●●●● ●●	acgtttgggtgtagcggtcgtcgggttggggtcggcgggagttcgcgggattttttagaagagcggtcggcgtcgtgatttagtattggggcggagcgg
1 unmethylated CpG in forward primer	●○●● ●●●● ●●	acgttcgggtgtagtggtcgtcgggttggggtcggcgggagttcgcgggattttttagaagagcggtcggcgtcgtgatttagtattggggcggagcgg
1 unmethylated CpG in forward primer	●●○● ●●●● ●●	acgttcgggtgtagcggttgtcgggttggggtcggcgggagttcgcgggattttttagaagagcggtcggcgtcgtgatttagtattggggcggagcgg
1 unmethylated CpG in forward primer	●●●○ ●●●● ●●	acgttcgggtgtagcggtcgttgggttggggtcggcgggagttcgcgggattttttagaagagcggtcggcgtcgtgatttagtattggggcggagcgg

Variant name	Variant methylation pattern	Sequence (5'- end to 3'-end)
	→ Forward 5' 3' → Probe 5' 3' ← Reverse 5' 3'	
2 unmethylated CpG's in forward primer	○○●● ●●●● ●●	acgtttgggtgtagtggtcgtcggggttggggtcggcgggagttcgcgggattttttagaagagcggtcggcgtcgtgatttagtattggggcggagcgg
2 unmethylated CpG's in forward primer	○●○● ●●●● ●●	acgtttgggtgtagcggttgtcggggttggggtcggcgggagttcgcgggattttttagaagagcggtcggcgtcgtgatttagtattggggcggagcgg
2 unmethylated CpG's in forward primer	○●●○ ●●●● ●●	acgtttgggtgtagcggtcgttggggttggggtcggcgggagttcgcgggattttttagaagagcggtcggcgtcgtgatttagtattggggcggagcgg
2 unmethylated CpG's in forward primer	●○○● ●●●● ●●	acgttcgggtgtagtggttgtcggggttggggtcggcgggagttcgcgggattttttagaagagcggtcggcgtcgtgatttagtattggggcggagcgg
2 unmethylated CpG's in forward primer	●○●○ ●●●● ●●	acgttcgggtgtagtggtcgttggggttggggtcggcgggagttcgcgggattttttagaagagcggtcggcgtcgtgatttagtattggggcggagcgg
2 unmethylated CpG's in forward primer	●●○○ ●●●● ●●	acgttcgggtgtagcggttgttggggttggggtcggcgggagttcgcgggattttttagaagagcggtcggcgtcgtgatttagtattggggcggagcgg
3 unmethylated CpG's in forward primer	○○○● ●●●● ●●	acgtttgggtgtagtggttgtcggggttggggtcggcgggagttcgcgggattttttagaagagcggtcggcgtcgtgatttagtattggggcggagcgg
3 unmethylated CpG's in forward primer	○●○○ ●●●● ●●	acgtttgggtgtagcggttgttggggttggggtcggcgggagttcgcgggattttttagaagagcggtcggcgtcgtgatttagtattggggcggagcgg
3 unmethylated CpG's in forward primer	○○●○ ●●●● ●●	acgtttgggtgtagtggtcgttggggttggggtcggcgggagttcgcgggattttttagaagagcggtcggcgtcgtgatttagtattggggcggagcgg
3 unmethylated CpG's in forward primer	●○○○ ●●●● ●●	acgttcgggtgtagtggttgttggggttggggtcggcgggagttcgcgggattttttagaagagcggtcggcgtcgtgatttagtattggggcggagcgg
4 unmethylated CpG's in forward primer	○○○○ ●●●● ●●	acgtttgggtgtagtggttgttggggttggggtcggcgggagttcgcgggattttttagaagagcggtcggcgtcgtgatttagtattggggcggagcgg
1 unmethylated CpG in reverse primer	●●●● ●●●● ●○	acgttcgggtgtagcggtcgtcggggttggggtcggcgggagttcgcgggattttttagaagagcggtcggcgtcgtgatttagtattggggtggagcgg
1 unmethylated CpG in reverse primer	●●●● ●●●● ○●	acgttcgggtgtagcggtcgtcggggttggggtcggcgggagttcgcgggattttttagaagagcggtcggcgttgtgatttagtattggggcggagcgg
2 unmethylated CpG's in reverse primer	●●●● ●●●● ○○	acgttcgggtgtagcggtcgtcggggttggggtcggcgggagttcgcgggattttttagaagagcggtcggcgttgtgatttagtattggggtggagcgg

Legend

Primer and probe target regions: sequences corresponding to the binding sites of primers and hydrolysis probe.

cg CpG site that is methylated (remains as cytosine after bisulfite conversion).

tg CpG site that is unmethylated (converted to uracil/thymine after bisulfite conversion).s
